# Individual characteristics of adolescent psychiatric patients accessing psychotherapy in China

**DOI:** 10.1038/s41598-022-21320-2

**Published:** 2022-10-09

**Authors:** Jinping Ma, Hai Zhou, Xinwei Li, Qinqin Fu, Guohua Lu

**Affiliations:** 1 School of Public Health, Weifang Medical University, Weifang, China; 2 Weifang Mental Health Center, Weifang, China; 3 School of Psychology, Weifang Medical University, Weifang, China

**Keywords:** Psychology, Human behaviour

## Abstract

Most mental health problems develop during childhood and adolescence, so identifying the mental health needs and care pathways of adolescents is crucial to improving prevention. This study aimed to understand the characteristics of adolescent patients with mental disorders receiving psychotherapy in China. Data were collected retrospectively from the psychotherapy records of 116 patients at the Weifang Mental Health Centre. Information collected included demographics, stressors, duration of psychotherapy, and clinical diagnosis. Chi-square tests and negative binomial regression models were used to explore the relationship between demographic and clinical variables. The results showed that depression was the most common diagnosis, followed by anxiety and stress-related disorders and bipolar and related disorders. Rural patients were more likely to report family stress, while urban patients were more likely to report school stress. Female patients were more likely to report family stress and to be diagnosed with depression. Family stress, social stress, school stress, patient origin and economic conditions were all significant predictors of the duration of psychological treatment. This study helps to understand the characteristics and psychotherapeutic needs of adolescents with mental disorders who receive psychotherapy in China so that the positive role of psychotherapy in the prevention, treatment and rehabilitation of mental disorders can be better utilised.

## Introduction

The issue of youth mental health in China has attracted a great deal of attention and focus. As far as the issue of youth mental health itself is concerned, it is also a significant problem commonly faced by countries worldwide. According to a new study by the American Psychological Association, reported by Xinhua on 18 March 2019, young people's mental health in the US has deteriorated^1^. In the twenty-first century, due to the rapid development and changes in society, more and more adolescents are experiencing severe psychological stress and distress. They are even experiencing severe mental depression or suicide attempts. The China Mental Health Development Report (2019–2020) shows that the mental health index of adolescents aged 12–18 is decreasing with age, and the detection rate of depression among adolescents is 24.6%, with severe depression reaching 7.4%^2^.

There is a growing consensus that early detection, prevention and intervention in adolescence can help reduce the burden of mental illness^3,4^. For this reason, understanding the characteristics of adolescent mental illness, perceived stressors and pathways of care is essential for early detection and treatment. Psychotherapy has proven to be an effective theoretical technique and is widely used in mental health and mental health promotion practice^5^. With the growing understanding of the biopsychosocial model of medicine, the causes of the disease have been elevated from a single consideration of biological factors to a holistic view, highlighting the impact of psychological, socio-environmental and natural environmental factors on human health. Studies at home and abroad have pointed out that the combination of pharmacological treatment and systematic psychological treatment can help patients with mental disorders improve their compliance with treatment^6,7^, which is of great importance in strengthening the assistance for patients with mental disorders, effectively reducing mental disability, promoting their rehabilitation and return to society, and maintaining social harmony and stability^7^. However, little research has been conducted in China on the population of adolescents seeking psychotherapy services. A small number of studies on adolescents have highlighted the high prevalence of psychiatric disorders^8,9^, including depression^10^ and anxiety disorders^11^. Reluctance to seek psychological help has also been reported due to stigma and skepticism about the efficacy of psychotherapy^12^. Although there is a paucity of literature examining the psychological treatment of adolescents in China, this literature highlights the high prevalence of mental health problems. It confirms the need for more excellent prevention and early intervention in the region. To this end, a better understanding of the role that factors play in developing symptoms and the choice to seek help is crucial.

Against this background, this study intends to quantitatively analyse the psychotherapy records of 116 patients to understand the characteristics and psychotherapy needs of young people with mental disorders receiving psychotherapy in China, and on this basis, propose a mental health service model that better meets the needs of patients, to better exploit the positive role of psychotherapy in the prevention, treatment and rehabilitation of mental disorders. The first goal of this study was to describe our sample by looking at demographic factors (gender and residential status), stressor types, frequency of self-harm and suicidality, and clinical diagnoses. The study's second goal was to look at the relationship between residential status, stressor frequency, and clinical diagnoses and the relationship between gender, stressor frequency, and clinical diagnoses. The third objective of this study was to find potential predictors of the duration of psychotherapy. Our study is the first attempt to describe the situation of adolescents with mental disorders receiving psychological treatment in China.

## Methods

### Study population and sample

This study was conducted in the psychotherapy outpatient clinic of a tertiary-level psychiatric hospital in Weifang. Retrospective data collection and variable extraction were conducted on psychotherapy records of adolescent patients in psychotherapy clinics between June 2019 and June 2021. The main techniques used in psychotherapy include cognitive therapy, supportive psychotherapy, and family therapy.

Psychotherapy records include information collected from adolescent patients and their families at treatment. Patients diagnosed with schizophrenia spectrum and other psychotic disorders, bipolar disorder and related disorders, depression, anxiety disorders, obsessive–compulsive disorder or trauma and stress-related disorders according to ICD-10 diagnostic criteria and aged between 12 and 18 years were included in the study. Patients were excluded if they also had a neurological disorder or if another severe disorder could explain their symptoms.

Data extracted from psychotherapy records included demographics (age, gender, residence, grade), presence of intentional self-harm and suicidal thoughts, primary diagnosis, and presence of psychotic features in the patient's symptoms.

As described in the case list, perceived life stressors that may trigger symptoms were divided into five categories: Family stress (e. g., conflict with a family member), Social stress (e. g. no friends), School stress (e. g. declining academic performance), Physiological stress (e. g. lack of sleep) and Spiritual stress (e. g. fear of death; see Table 1 for more details). Each category was treated as a dichotomous variable (yes = 1; no = 0). The duration of psychotherapy includes the duration of treatment in terms of the number of sessions (i.e. the time from the first psychotherapy session to the end of treatment).

**Table 1:** Classification of stressors and illustrations.

Stressors	Illustrations
Family stress	Parental divorce, parental quarrel, the family member with mental illness, death of a family member, birth of a family member, conflict with a family member, poor family financial situation
Social stress	School bullying, sexual harassment, no friends, social isolation, friends leaving, death of friends, conflict with friends, relationship problems
School stress	Learning difficulties, dissatisfaction with school management style, not wanting to go to school, declining academic performance, failing exams, poor teacher relationship, and going to a new school
Physiological stress	Poor appetite, lack of sleep, frequent headaches, puberty, gastrointestinal problems, recent surgery, exercise problems
Spiritual stress	Fear of death, guilt, lack of confidence

### Data analysis

Statistical analyses were performed using SPSS version 26. Frequency analysis was used to describe our sample's demographic and clinical characteristics and the frequency of stressors experienced. The Mann–Whitney U test was used to compare the psychotherapy duration of different genders. Chi-square tests were performed to investigate the impact of demographic differences on the stressors faced by adolescent patients and clinical diagnoses. Regression models using count data were used to identify predictors of psychotherapy duration (i.e. number of sessions). Violations of the equidiscrete assumption were assessed following Payne et al.^13^; the Chi-square/df ratio indicated overdispersion. Therefore, a negative binomial regression model was chosen based on Poisson^13^. The model's potential predictors of psychotherapy duration included family stress, social stress, school stress, depressive disorders, anxiety and stress-related disorders, bipolar disorder, patient origin, economic conditions, self-harm, and suicidality.

### Ethics approval and consent to participate

Weifang Medical University Ethics Committee has granted ethics approval (project ID: 2022YX043). Participants and their legal guardians gave verbal and electronic informed consent to participate in this study. This study adheres to the ethical principles set out by the World Medical Association WMA through the Declaration of Helsinki (1996 edition, revised 2013), the International Ethical Guidelines for Biomedical Research Involving Human Beings (2002) in collaboration with the International Council for Medical Sciences (CIOMS) of the World Health Organization (WHO). All methods were performed following relevant guidelines and regulations.

## Results

The initial sample consisted of 146 patients. Thirty of these were later excluded, either because they did not meet the inclusion criteria or because the psychotherapy record form contained insufficient information. The final sample consisted of 116 patients, and the demographic characteristics of the sample are shown in Table 2.

**Table 2:** Demographic characteristics.

Variable	*N* = 116	
Age (years)	Mean (SD)	
	15.6 (1.5)	
**Sex**	Frequency (%)	95% CI
Male	39 (33.6%)	24.9–42.3
Female	77 (66.4%)	57.7–75.1
**Place of residence**		
Urban	74 (63.8%)	54.9–72.7
Rural	42 (36.2%)	29.3–45.7
**Patient origin**		
Outpatient	86 (74.1%)	66.0–82.2
Ward	30 (25.9%)	17.8–34.0
**Diagnosis**		
Depressive disorders	57 (49.1%)	39.9–58.4
Anxiety disoders, obsessive compulsive disorder and stress related disorders	43 (37.1%)	28.1–46.0
Bipolar and related disorders	10 (8.6%)	3.4–13.8
Schizophrenia spectrum and other psychotic disorders	6 (5.2%)	1.1–9.3

N, number; SD, standarddeviation; CI, confidence interval.

In terms of the categories of stressors in the overall sample, family stress was the most frequently reported (62.9%), followed by social stress (37.1%), school stress (38.8%), and physiological stress (10.3%) and spiritual stress (6.9%).

Table 3 presents the frequency of stressors experienced by place of residence (urban/rural) and gender. Rural patients had a higher frequency of family stress than urban patients (73.8% versus 54.1%; *χ*^2^ = 4.40; *P* < 0.05).

**Table 3:** Frequency of stressors by residential status and gender.

Stressors	Residential status% (95%CI)		Gender% (95%CI)	
	Urban *n* = 74	Rural *n* = 42	Male *n* = 39	Female *n* = 77
Family stress	54.1 (42.4, 65.7)	73.8 (59.9, 87.7)	53.8 (32.3, 65.1)	72.7 (56.8, 78.2)
Social stress	40.5 (29.1, 52.0)	30.0 (16.4, 45.5)	38.5 (22.5, 54.4)	36.4 (24.4, 47.4)
School stress	48.6 (37.0, 60.3)	21.4 (8.5, 34.4)	30.8 (15.6, 45.9)	42.9 (31.6, 45.2)
Physiological stress	9.5 (2.6, 16.3)	11.9 (1.7, 22.1)	7.7 (0.0, 17.8)	11.7 (4.3, 19.0)
Spiritual stress	6.8 (0.9, 12.6)	7.1 (0.0, 15.3)	6.5 (0.9, 12.1)	

*n*, number; CI, confidence interval.

Urban patients were more likely to report school stress than rural patients (48.6% vs 21.4%; *χ*^2^ = 8.36; *P* < 0.05). No between-group differences were observed in the frequency of social stress between urban and rural patients (40.5% and 30.0%, respectively). However, this difference did not reach statistical significance, possibly because of the small sample size. In terms of gender, females experienced family stress more frequently than males in our sample (males: 53.8%; females: 72.7%; *χ*^2^ = 4.14; *P* < 0.05); there were no significant gender differences in the frequency of school stress (males: 30.8%; females: 42.9%) or social stress (males: 38.5%; females: 36.4%). The relationship between gender, residential status and physical and spiritual stress was not calculated, as only a minority of patients reported these stressors.

Depression was diagnosed in 49.1% of patients. Of these patients, 57.9% (*n* = 33) reported self-harm and 19.3% (*n* = 11) reported suicidality. 8.6% were diagnosed with bipolar and related disorders. None of these patients had self-harm; 20% reported suicidality (*n* = 2). 37.1% of patients were diagnosed with anxiety, obsessive–compulsive, trauma and stress-related disorders. 2.3% (*n* = 1) reported self-harm and no suicidality among these patients. Only 5.2% of patients were diagnosed with schizophrenia spectrum and other psychiatric disorders; these patients were excluded from the analysis conducted for each diagnostic group due to the small sample size. 16.7% of patients (*n* = 1) reported self-harm and suicidality.

In terms of the relationship between clinical diagnosis and residential status, there was no significant difference between urban and rural patients in terms of the prevalence of depressive disorders (*χ*^2^ = 1.04; *P* = 0.31), anxiety and related disorders (*χ*^2^ = 0.39; *P* = 0.53), bipolar disorder and related disorders (*χ*^2^ = 2.68; *P* = 0.10) or schizophrenia (*χ*^2^ = 2.54; *P* = 0.11) differences. Female helpers were more likely to suffer from depression than males (*χ*^2^ = 5.87; *P* < 0.05). There was no significant difference between males and females in the prevalence of anxiety disorders, bipolar disorder and related disorders (anxiety plus obsessive–compulsive: *χ*^2^ = 3.42; *P* = 0.07; bipolar *χ*^2^ = 1.32; *P* = 0.25; schizophrenia: *χ*^2^ = 0.00; *P* = 0.99).

The negative binomial regression results for psychotherapy duration indicated that family stress was a significant predictor of psychotherapy duration (IRR = 0.57; 95% CI = 0.34–0.96; p < 0.05). Specifically, those who did not report family stress were 0.57 times more likely to have a duration of psychotherapy compared to those who reported family stress. Similarly, treatment duration was significantly associated with social stress (IRR = 0.63; 95% CI = 0.40–0.99; *p* < 0.05), indicating that psychotherapy lasted 0.63 times longer for those who did not report social stress compared to those who reported social stress. Treatment duration was significantly associated with school stress (IRR = 0.62; 95% CI = 0.38–1.00; *p* < 0.05), indicating that psychotherapy duration was 0.62 times longer for those who did not report school stress compared to those who reported school stress. In addition, the duration of psychotherapy was significantly associated with the patient source, indicating that psychotherapy duration was 0.48 times longer for outpatients than for inpatients (IRR = 0.48; 95% CI = 0.28–0.82; *p* < 0.05). Finally, household economic status was significantly associated with the duration of psychotherapy (IRR = 0.58; 95% CI = 0.35–0.94; *p* < 0.05); those patients who reported average economic conditions had a duration of psychotherapy that was 0.58 times longer than those with good economic conditions. These results are presented in Table 4.

**Table 4:** Results of the negative binomial regression.

Predictor*	B	SE	Wald Chi-Square	*P*	IRR	95%CI
Family stress	− 0.57	0.27	4.61	*p* < 0.05	0.57	0.34–0.96
Social stress	− 0.46	0.27	3.75	*p* < 0.05	0.63	0.40–0.99
School stress	− 0.48	0.24	3.9	*p* < 0.05	0.62	0.38–1.00
Depression	− 0.08	0.43	0.04	0.84	0.92	0.39–2.14
Anxiety	− 0.39	0.39	0.99	0.32	0.68	0.32–1.46
Bipolar	− 0.36	0.49	0.52	0.47	0.7	0.27–1.84
Source of patients	− 0.74	0.28	7.22	*p* < 0.05	0.48	0.28–0.82
Economic situation	− 0.55	0.26	4.65	*p* < 0.05	0.58	0.35–0.95
Suicidality	− 0.04	0.39	0.01	0.92	0.96	0.45–2.07
Self-harm	− 0.47	0.35	1.73	0.19	0.63	0.31–1.26

*All predictors compare No = 0 versus Yes = 1 except Economic situation and Source of patients where General = 0 and Better = 1, outpatient = 0 and ward = 1.

*B,* regression coefficient; SE, standard error; *P*, *p*-value; IRR, rate ratio; CI, confidence interval.

## Discussion

This study highlights several key findings. First, we observed a high prevalence of depression in a sample of adolescents seeking psychological treatment, consistent with previous studies conducted in China^14,15^. In addition, patients diagnosed with depression appeared to have the highest frequency of self-harm and suicide compared to other diagnostic groups, consistent with reports in the national and international literature^16–19^. Studies have shown that mental disorders are strongly associated with suicide, with depression being the most common psychiatric disorder causing suicide in men and women^17,20^. In addition, self-injury is common in young people with mental ill-health. However, it is widespread in individuals with mood disorders^21–23^, and there is a significant association between mood dysregulation and self-injury^24^. Research has shown that poor emotional regulation in depressed adolescents is the main reason for the high prevalence of self-harm^25^. When individuals cannot deal with negative emotions properly, self-injurious behaviour becomes an outlet for venting bad feelings^26^.

The prevalence of family stress across the sample confirms the well-known link between poor family relationships and adolescent mental health trajectories^27–30^. Studies have shown that chronic or intense stress can lead to high prefrontal dopamine concentrations, resulting in impaired functioning and increased emergence of psychotic symptoms and mood disorders^31,32^. Adverse family environments can act as a stressor, leading to dysregulation of adolescents' biological stress response system. Studies have found that children who experience early adverse care exhibit impaired psychological development^33^.

Rural adolescent patients were more likely to cite family relationships as a source of stress, while urban adolescent patients reported more frequently than those stressed about school-related issues. Studies have found that rural adolescents are significantly less likely than urban adolescents to have lower family closeness and family member communication^34^. It is consistent with the findings of our study. It may be due to the reality that parents need to seek development outside the home in rural areas due to the more underdeveloped economy, leading to reduced contact between children and parents, barriers to communication between family members and reduced levels of family closeness. In contrast, urban adolescents face more academic pressure. This may be due to the high proportion of only children in urban areas in the context of China's unique family planning population policy and quality education and culture, where high parental expectations drive greater emphasis on their children's academic performance and as a means of achieving family honour^35^.

Females report family stress more frequently than males; this finding is consistent with a previous study on family functioning and help-seeking behaviour. On the one hand, in China, where there is a long-standing traditional concept of passing on the family line and continuing the family tradition, and the preference for male children has led to gender discrimination within the family, the gender preference for sons over daughters leads to gender discrimination within the family^36^, and this tangible or intangible pressure can harm women's mental health. On the other hand, Adolescent females were more likely to report high conflict levels within the family than males^37^. Some studies have shown that girls are more sensitive to cultural trends and social changes than boys^38^, feel more prominently about negative emotions and events and that external environmental factors have a more significant impact on girls^39^. It points to the possibility that female students may be particularly vulnerable to psychiatric decompensation^40^.

Among our adolescents seeking psychological treatment, depression is more common in female patients than in male patients. It has been confirmed in the literature^41–43^ and may reflect gender differences in depression in the psychotherapy-seeking population^42,43^. Meta-analyses have confirmed that the prevalence of depression is approximately twice as high in women as in men. Women are more likely than men to suffer from adverse social and material influences to develop depression^44^. It may be related to disturbances in the HPA axis (elevated cortisol, HPA-negative feedback inhibition) that differ between genders with depression^45^. It is also associated with differences in depression-related biological markers between the sexes, and for example, women show higher levels of inflammation^46^ and neurotrophic and serotonergic markers^47,48^. Fluctuations in oestrogen levels are associated with the pathogenesis of depression in women, who are more likely to experience depression during periods of hormonal change such as puberty^49^.

The regression analysis results in this study showed that adolescent patients who reported family stress, social stress, and school stress had a longer duration of psychotherapy than those who did not. First, the prevalence of family stress in our sample and the fact that parents are often the primary initiators of the psychotherapeutic process are not unexpected results. Research has shown that adolescents are more likely to seek relief from their families when experiencing psychological problems. Poor parent–child relationships and harmful parenting practices can increase adolescent health-seeking behaviour^50^. Second, experiences of social-related stress often include having no friends or suffering from isolation. In our sample, a lack of social support may have led to a prolonged duration of psychological treatment. We suggest that groups of adolescents are prone to psychological distress but are reluctant to seek help from friends and teachers and more likely to seek help from psychologists, leading to a prolonged duration of psychological treatment. Finally, school-related stress experiences often include learning difficulties and dissatisfaction with the school management system. In recent years, adolescents have been burdened with excessive academic stress due to increased academic burdens and great parental expectations^51^, and academic stress can hurt healthy lifestyles and mental health.

Finally, inpatients and better family financial conditions predict a longer duration of psychological treatment, which has been less studied. Firstly, Inpatients are prone to rebelliousness and resistance to medical advice such as taking medication due to factors such as closed treatment, while research has shown that psychotherapy can significantly improve patients' compliance with treatment, help them eliminate their sense of shame and inferiority and restore their social functioning^52^. Therefore, mental health workers should be fully aware of the limitations of medication and the importance of psychological treatment, and strive to improve psychological treatment capabilities. Secondly, the better the financial situation of the patient's family, the longer the duration of psychotherapy, which means that professional services such as psychotherapy are difficult for low-income people to access and maintain, a pattern that has been confirmed in the literature^53,54^. This finding also highlights the need for an orderly inclusion of outpatient psychotherapy costs in health insurance payments^55^. Although some cities and regions in China have included mental disorders in their medical insurance as outpatient chronic illnesses, the vast majority of patients are reimbursed for the cost of medication and tests. However, unlike the relatively manageable expenditure on medication, psychological treatment is often a more important expense for young people with mental disorders, and the cost of treatment has become a major factor limiting treatment for people with mental illness. For this reason, Zhu Jinsong, a deputy to the National People's Congress, put forward a proposal for the orderly inclusion of psychological treatment in the scope of medical insurance payments.

## Strengths, limitations, and future research

To our knowledge, this is the first study to attempt to characterize adolescent psychiatric disorder patients receiving psychotherapy services in China. However, our sample was relatively small and selective, but it is important to note that the data were obtained from psychotherapy files of a tertiary psychiatric hospital in China. Another limitation of the current work is that it is mainly based on self-reported information (collected from patients or family members). Therefore, our data may lack objectivity or some relevant information (e.g., substance abuse) may have been omitted due to its legal significance.

The future development direction is in line with the global health action to improve, and the Chinese government attaches great importance to the nation's mental health. On 25 October 2016, the Central Committee of the Communist Party of China (CPC) and the State Council issued and implemented the “Health China 2030” Planning Outline to promote the construction of a healthy China and improve people's health, to propose to increase the popularisation of mental health science for all people and improve mental health literacy. Interventions for common mental disorders such as depression and anxiety disorders and psychological and behavioural problems will be strengthened, and early detection and timely intervention for psychological problems in crucial populations will be stepped up. By 2030, the prevention and treatment of common mental disorders and identification and intervention of psychological and behavioural problems will be significantly improved. Since then, significant progress has been made in recognising, awareness, and treating mental disorders. However, some recommendations for the future can be made. Developing basic research on mental health issues, particularly efficacy and localised research, will help to improve the professionalism and relevance of psychological treatment in China; doing an excellent job in popularising mental health-related science will help to improve public discernment and create a social environment in which psychological counselling and treatment teams are superior and inferior.

## Conclusions

This study helps to describe the characteristics of adolescents with psychiatric disorders receiving psychotherapy in China. A high prevalence of depression was found in our sample, and a prevalence of poor family functioning was observed. Gender differences in perceived stressors and clinical diagnoses, possibly related to the economic level and cultural norms, suggest the need to promote help-seeking behaviour in adolescent girls further. Those who reported family stress and social and school stress lasted longer in psychotherapy. In addition, psychotherapy lasted longer for adolescents whose families were better off financially. Overall, this study helps to understand the characteristics and psychotherapeutic needs of adolescents with mental disorders who receive psychotherapy in China so that the positive role of psychotherapy in the prevention, treatment and rehabilitation of mental disorders can be better utilised. It may help plan targeted prevention strategies to improve the life trajectories of adolescents living in China in line with the government's strategic plan.

## Data Availability

Due to the sensitive and confidential nature of patients' psychotherapy records, which may reveal the identity of participants, the data sets generated and analysed in the current study are not publicly available. However, they can be obtained from the corresponding authors upon reasonable request.
